# Investigating the association between dental age and polymorphisms in genes encoding estrogen receptors

**DOI:** 10.1590/1678-7757-2023-0184

**Published:** 2023-09-25

**Authors:** Isabela Ribeiro MADALENA, Caio Luiz Bitencourt REIS, Mirian Aiko Nakane MATSUMOTO, Maria Bernadete Sasso STUANI, Natanael Henrique Ribeiro MATTOS, Daniela Silva Barroso de OLIVEIRA, Maria Angélica Hueb de Menezes OLIVEIRA, Liliane ROSKAMP, Erika Calvano KÜCHLER, Flares BARATTO-FILHO

**Affiliations:** 1 Universidade da Região de Joinville Departamento de Odontologia Joinville Santa Catarina Brasil Universidade da Região de Joinville - UNIVILLE, Departamento de Odontologia, Joinville, Santa Catarina, Brasil.; 2 Universidade de Uberaba Departamento de Biomateriais Uberaba Minas Gerais Brasil Universidade de Uberaba - UNIUBE, Departamento de Biomateriais, Uberaba, Minas Gerais, Brasil.; 3 Centro Universitário Presidente Tancredo de Almeida Neves UNIPTAN Faculdade de Odontologia São João del Rei Minas Gerais Brasil Centro Universitário Presidente Tancredo de Almeida Neves - UNIPTAN, Faculdade de Odontologia, São João del Rei, Minas Gerais, Brasil.; 4 Universidade de São Paulo Faculdade de Odontologia de Ribeirão Preto Departamento de Clínica Infantil Ribeirão Preto São Paulo Brasil Universidade de São Paulo, Faculdade de Odontologia de Ribeirão Preto, Departamento de Clínica Infantil, Ribeirão Preto, São Paulo, Brasil.; 5 Universidade Tuiuti do Paraná Curitiba Paraná Brasil Universidade Tuiuti do Paraná - UTP, Curitiba, Paraná, Brasil.; 6 Universidade Federal de Alfenas Faculdade de Odontologia Departamento de Clínica e Cirurgia Minas Gerais Brasil Universidade Federal de Alfenas, Faculdade de Odontologia, Departamento de Clínica e Cirurgia, Minas Gerais, Brasil.

**Keywords:** Odontogenesis, Estrogen, Genes

## Abstract

**Background:**

Genetic polymorphisms have been shown to influence several physiological traits, including dental and craniofacial characteristics. Understanding the clinical relevance of genetic polymorphisms in dental practice is crucial to personalize treatment plans and improve treatment outcomes.

**Objective:**

to evaluate the association between dental age and genetic polymorphisms in genes encoding estrogen receptors alpha and beta (*ESR1* and *ESR2*, respectively) in a sample of Brazilian children.

**Methodology:**

This retrospective cross-sectional study was performed with children undergoing orthodontic treatment. Patients with syndromes, congenital anomalies, craniofacial deformities, under hormonal or systemic treatment, and with a previous history of facial trauma were excluded. Panoramic radiographs were used to assess dental age according to the Demirjian, Goldstein, and Tanner method. A delta [dental age-chronological age (DA-CA)] was obtained, which shows whether the patient tends to have a normal, delayed (negative values), or advanced (positive values) dental age. DNA isolated from buccal cells was used to genotype four genetic polymorphisms: rs9340799 (A>G) and rs2234693 (C>T), located in *ESR1*; and rs1256049 (C>T) and rs4986938 (C>T), located in *ESR2*. A statistical analysis was performed and values of p<0.05 indicated statistical difference.

**Results:**

A total of 79 patients were included, 44 (55.70%) girls and 35 (44.30%) boys. The Demirjian, Goldstein, and Tanner method, in general, overestimated patients’ age by 0.75 years. There was no difference in the delta of dental age between the sexes (p>0.05). Genetic polymorphisms in ESR1 and ESR2 were not associated with dental age (p>0.05).

**Conclusion:**

The studied genetic polymorphisms in *ESR1* and *ESR2* were not associated with dental age in Brazilian children.

## Introduction

Tooth development begins in humans around the eighth week of pregnancy and lasts until approximately 18 years of age.^1^ Tooth development is a long and complex process that occurs synchronously with several important episodes in the child’s growth and development.^2,3^ Knowledge of the aspects involved in tooth development is important in clinical practice,^4,5^ forensic practice,^6^ and anthropology.^7^ Several studies estimate chronological age by analyzing the stages of tooth development.^8-10^ The most commonly used methods to assess dental age are Nolla^11^ (1960); Cameriere, Ferrante, and Cingolani^12^ (2006); and Demirjian, Goldstein, and Tanner^13^ (1976). Although dental age methods are a common index to determine chronological age, there is a great individual variation observed in each study.

Many aspects may be involved in the individual variation of dental age. Several factors play an important role in tooth development, such as local, environmental, and systemic factors, including hormones and genetic factors.^14-16^ It is estimated that more than 300 genes are expressed during the process of tooth development,^17-20^including genes coding for hormones and hormones receptors. Recent studies point to the presence of the main estrogen receptors in dental tissues. The receptors ERα (estrogen receptor alpha) and ERβ (estrogen receptor beta), encoded by the *ESR1* and *ESR2* genes, respectively,^21^ have been observed in the odontogenic region of teeth^19,20^ and with osteogenic potential in pulp cells in human teeth.^22,23^

There is also evidence that estrogen is involved in changes in tooth development time and dental maturity, which was observed in clinical^24^ and animal model studies.^20^ Moreover, genetic polymorphisms in *ESR1* and *ESR2* have been associated with maxillary and mandibular growth phenotypes^25^ and tooth size.^26^ It is possible that *ESR1* and *ESR2* also play a role in tooth development and affect dental age. The identification of specific genetic markers associated with tooth development will enable personalized treatment plans to maximize the efficiency and predictability of dental and orthodontic interventions for each patient. Genetic polymorphisms have also been associated with an increased risk of developing tooth disorders, such as delayed tooth eruption,^27^ primary failure of eruption,^28^ among others. Therefore, in this study, we investigated whether genetic polymorphisms in *ESR1* and *ESR2* are associated with delayed or advanced dental age in a sample of Brazilian children.

## Methodology

### Ethical aspects

This project was approved by the Human Research Ethics Committee of the School of Dentistry of Ribeirão Preto, University of São Paulo (FORP/USP) (CAAE #01451418.3.0000.5419). Informed consent was obtained from all participants and/or their legal guardian.

### Sample characterization

This is a cross-sectional phenotype-genotype study with children aged seven to 16 years undergoing orthodontic treatment at the School of Dentistry of Ribeirão Preto, University of São Paulo (FORP/USP), from 2015 to 2017. Orthodontic records of children of both sexes were screened. Patients with syndromes, congenital anomalies, craniofacial deformities, under hormonal or systemic treatment, and with a previous history of facial trauma were excluded.

The sample size was estimated using G*Power Version 3.1.9.6 (Franz Faul, Universität Kiel, Germany). The difference between two independent means was measured, with alpha equal to 5% and 80% power. The effect size (Cohen’s D=0.72) was obtained from Hilgers, et al. (2006). The calculation predicts a minimum of 77 patients for the sample of this study, considering a loss rate of 20%.

### Phenotype definition – Tooth development/dental age analysis

Dental age was assessed according to the Demirjian, Goldstein, and Tanner method.^13^ The degree of maturation of each permanent tooth on the left side of the mandible (excluding the third molar) was assigned. The seven left mandibular molars were scored: 0 for no calcification and A to H according to the stage of calcification of the tooth. The scores for boys and girls were converted into weighted scores according to sex. Dental age (DA) was then estimated using maturity charts and the value obtained was the DA according to the Demirjian, Goldstein, and Tanner method.^13^ In the case of tooth agenesis/missing tooth on the left side, the contralateral permanent tooth on the right side was evaluated. The child was excluded from the study if one or more bilateral teeth were missing.

A delta [dental age-chronological age (DA-CA)] was obtained, which shows whether the patient tends to have a normal, delayed (negative values), or advanced (positive values) dental maturity, in line with a previous study.^29^ Two observers trained by a senior orthodontist were previously trained and calibrated. The weighted Cohen’s Kappa test was performed for each tooth evaluated. Intraobserver reliability ranged from 0.82 to 1.00 and interobserver reliability ranged from 0.79 to 1.00.

### Genotyping analysis

Genomic DNA for molecular analysis was extracted from saliva cells using the method described by Küchler, et al.^30^ (2012). Four intronic genetic polymorphisms with a minor allele frequency of more than 20% were selected, based on previous studies.^25,26^ The selected genetic polymorphisms rs9340799 (A>G) and rs2234693 (C>T) are located in *ESR*1, and rs1256049 (C>T) and rs4986938 (C>T) are located in *ESR2*. The laboratory experiment was performed blinded to the patient’s condition. Genotyping was performed by real-time polymerase chain reactions (real-time PCR), using the TaqMan assay, StepOnePlus Real-Time PCR System (Applied Biosystems, Foster City, California, USA).

### Statistical analysis

Dental age (delta DA-CA) was assessed as a continuous variable. Chi-square was used to estimate the Hardy-Weinberg equilibrium. Statistical analysis was performed using GraphPad Prism 9 and Plink. The Mann-Whitney test was used to compare DA between sexes. The Kruskal-Wallis or Mann-Whitney test were used to compare dental age according to genotypes. A linear regression analysis was performed using sex as a covariable. Haplotype analysis was also performed. For all the analyses in this study, statistical significance was established at 5%.

## Results

**Figure 1:**
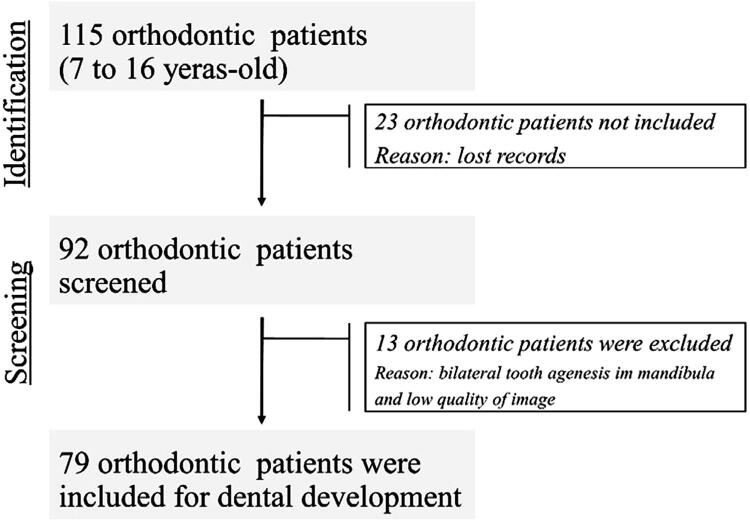
Flowchart of the sample selection process and outcome

A total of 115 orthodontic patients were screened, 79 of whom were included in this study according to the inclusion/exclusion criteria (Figure 1).

**Figure 2:**
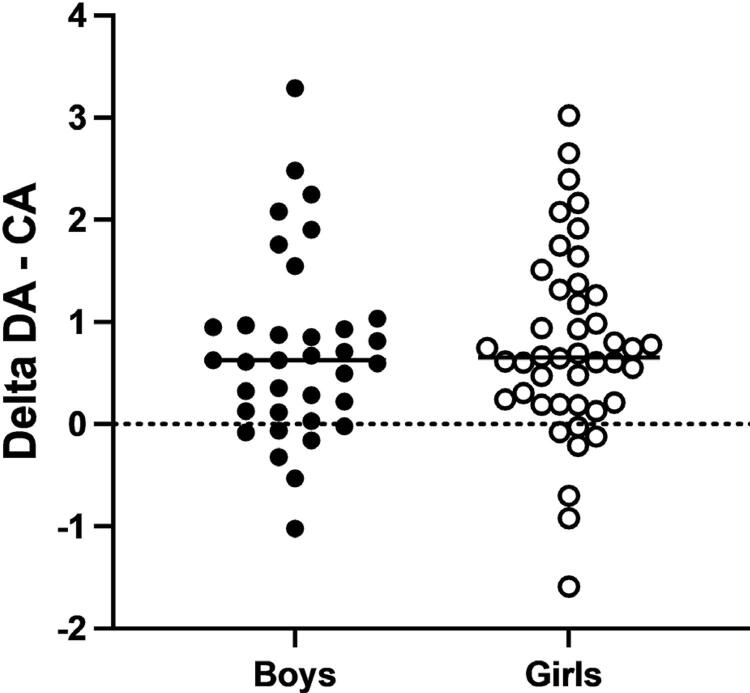
Distribution of delta (DA-CA) according to sex. The mean delta for boys was 0.72 (standard deviation=0.90), while the mean delta for girls was 0.77 (standard deviation=0.91)

In total, 44 (55.7%) patients were girls and 35 (44.3%) patients were boys. The Demirjian, Goldstein, and Tanner method^13^ overestimated the age of patients by 0.75 years. Figure 2 shows the comparison of delta DA-CA between the sexes. There is no statistical difference (p=0.676).

The genetic polymorphisms studied were in Hardy-Weinberg equilibrium (p>0.05). Delta DA-CA was compared between the genotypes of each genetic polymorphisms studied (Table 1), and no genetic polymorphism was associated with dental age. The linear regression performed according to sex for genotypic (Table 2) and allelic distribution (Table 3) also showed that genetic polymorphisms in the *ESR1* and *ESR2* genes were not associated with dental age.

**Table 1:** Comparison of delta DA-CA between genotypes

Genetic polymorphims	Genotype	n	Median	25^**th**^ quartile	75^**th**^ quartile	p-value
rs9340799	AA	40	0.647	0.226	1.303	0.467#
	AG	30	0.619	0.016	0.934	
	GG	6	0.400	-0.088	0.997	
rs2234693	CC	12	0.636	-0.0007	1.021	0.877#
	CT	33	0.643	0.052	1.162	
	TT	31	0.613	0.221	1.264	
rs1256049	CC	0	-	-	-	-
	CT	6	-0.079	0.193	1.945	0.593*
	TT	73	-1.588	0.187	1.01	
rs4986938	CC	23	0.626	0.126	1.177	0.521#
	CT	40	0.652	0.198	0.946	
	TT	11	0.306	-0.064	0.967	

NOTE: #Kruskal-Wallis test, -excluded from the analysis, *Mann-Whitney test was used.

**Table 2:** Linear regression analysis adjusted by sex

Variable (Genotype)	Estimate	SE	95% CI	t	*p*- value
^#^rs2234693 [CT]	-0.096	0.356	-0.808 to 0.616	0.269	0.788
^#^rs2234693 [CC]	-0.375	0.556	-1.487 to 0.735	0.675	0.501
*rs9340799 [AG]	-0.195	0.353	-0.901 to 0.510	0.554	0.581
*rs9340799 [GG]	0.016	0.654	-1.292 to 1.324	0.024	0.980
rs1256049 [CT]	0.032	0.449	-0.867 to 0.932	0.072	0.942
^$^rs4986938 [CC]	-0.194	0.271	-0.737 to 0.348	0.715	0.477
^$^rs4986938 [TT]	-0.339	0.335	-1.010 to 0.331	1.011	0.315

Note: SE means standard error, CI means confidence interval.

^#^TT was a reference; *AA was a reference; and ^$^CT was a reference.

**Table 3:** Linear regression analysis for allele distribution

Polymorphim	Estimate	SE	T	*p*-value
rs9340799	-0.156	0.129	-1.211	0.229
rs2234693	-0.045	0.130	-0.351	0.725
rs1256049	0.310	0.335	0.925	0.357
rs4986938	-0.091	0.138	-0.663	0.509

Note: SE means standard error.

Haplotype analysis was also performed within genetic polymorphisms in *ESR1* (rs9340799 and rs2234693) and *ESR2* (rs1256049 and rs4986938) and also showed no association (Table 4).

**Table 4:** Haplotype analysis

Chromosome	Haplotype	Haplotype order	Estimate	*p*-value
6	GC	rs9340799|rs2234693	-0.159	0.230
6	AC	rs9340799|rs2234693	-0.039	0.834
6	AT	rs9340799|rs2234693	0.159	0.189
14	CT	rs1256049|rs4986938	-0.091	0.509
14	TC	rs1256049|rs4986938	0.066	0.855
14	CC	rs1256049|rs4986938	0.081	0.556

## Discussion

Understanding the mechanisms involved in the process of tooth development and the factors that affect dental age is fundamental in the health sciences and can help clarify the factors involved in individual differences between chronological age and dental age. The mechanisms involved in tooth development are still largely unknown. Only a few studies have investigated the role of genetic polymorphisms in dental age in humans.^29,31^ Understanding the relationship between dental age and genetic polymorphisms can have significant implications for clinical practice. Dental age assessment is a crucial aspect of orthodontic treatment planning, as it helps determine the optimal timing and approach for interventions. Identifying the genetic factors that may influence dental age can enable more precise treatment strategies for each patient.

Estrogen and its receptors have been extensively studied in dental research in recent years due to their physiological importance for many vital tissues and organs and for the pubertal development of girls and boys.^19,20,25,26,32-34^ To the best of our knowledge, this is the first study to evaluate the association between genetic polymorphisms in *ESR1* and *ESR2* and tooth development/dental age. Our results show that dental age variability is not associated with the studied genetic polymorphisms in *ESR1* and *ESR2* in a sample of Brazilian children.

To assess dental age, the Demirjian, Goldstein, and Tanner method^13^ was used. This method estimates dental age according to the stage of dental calcification and is widely used in dental practice, for developmental analysis, and in forensic practices in different populations.^8-10,19,35^ It has an advantage over other techniques, as it does not need require radiographic complementation. The Demirjian, Goldstein, and Tanner method^13^ uses panoramic radiographs that are routinely used in clinical practice. In our study, the panoramic radiographs were taken from preorthodontic records of patients requiring orthodontic treatment. Our results showed that the Demirjian, Goldstein, and Tanner method^13^ overestimated the dental age of the children in this sample by a few months. Although this discrepancy between dental age and chronological age may be clinically relevant, it is important to highlight that the overestimation was only by a few months. It is worth mentioning that the Demirjian, Goldstein, and Tanner method^13^ was developed for the assessment of French-Canadian children, but the method was chosen due to its satisfactory performance in Brazilian children.^10^ It is possible that the genetic background is involved in the variation between regions.^36^

The difference between dental age and sex can be found in studies using large populations.^37^ In our study, there was no statistical difference between the sexes. However, it is important to note that the Demirjian, Goldstein, and Tanner method^13^ consider sex when estimating dental age; therefore, our results support that the method performs similar for both sexes. In a Spanish study that included children and adolescents aged seven to 21 years, the children were divided into three age groups: under 14, 14 to 18, and over 18.^38^ The dental age of both sexes was also overestimated and showed slight differences between the sexes depending on the age group evaluated.^38,39^ Children and adolescents under 14 years of age showed slight differences between sex and dental age. These results delimit a group of individuals at an intense stage of development. The Demirjian, Goldstein, and Tanner method^13^ suggests an age limit of three to 16 years. The lowest age that can be assessed is a limit of 16 years for the complete rooting of permanent second molars. They are usually the last teeth to emerge in the oral cavity, and root formation is completed at around 14 years and nine months of age for girls and 15 years and five months for boys.^40^

It is essential to mention the differences between the development of boys and girls due to the interaction of sex hormones, which are also reflected in oral tissues. Scientific evidence shows that changes in serum estrogen levels can affect the growth of the maxilla and mandible,^41^ tooth eruption,^20^ gene expression in the odontogenic region, and even the morphology of tooth structure.^19^ The estrogen signaling pathway is mainly performed by its main receptors (ERα and ERβ), encoded by *ESR1* and *ESR2*.^21^ Thus, we suggest the hypothesis that polymorphisms in *ESR1* and *ESR2* could affect tooth development in Brazilian children and be associated with variability in dental age. It is known that the level structure of a protein and its expression are influenced by genetic polymorphisms.^42^

Among the many polymorphisms in *ESR1*, the two most studied are rs2234693 (also known as PvuII or 397T>C) and rs9340799 (also known as XbaI or 351A>G). Genetic polymorphisms in rs2234693 and rs9340799 have already been described in association with tooth size.^26^ The authors also suggest that this finding is the result of changes resulting from tooth development. Regarding polymorphisms in *ESR2* (rs1256049 and rs4986938), tooth agenesis was also associated with rs4986938.^43,44^ Odontogenesis is under strict molecular control,^44^ thus, an alteration in different genes/molecules can lead to tooth agenesis. The authors also suggest complementing the scientific evidence to evaluate the expression of estrogen receptors during the stages of odontogenesis. Although our results showed no statistically significant difference between genetic polymorphisms in *ESR1* and *ESR2* and dental age variability, it is possible that other genes or genetic polymorphisms in these genes are involved in dental age. Modesto, et al.^31^ (2019) assessed whether genetic polymorphisms in growth factors (*IGF, FGFs,* and *FGFRs*) were involved in dental age and observed that *FGF18* (rs4073716) was associated with an older dental age than the child’s chronological age. Genetic polymorphisms in the vitamin D receptor (VDR) were not associated with dental age in the study by Küchler, et al.^29^(2022), also in Brazilian children.

Although no direct association was identified between the genetic polymorphisms studied and dental age, our study is of clinical relevance, as it sheds light on the complex genetic mechanisms involved in tooth development. As we continue to expand our knowledge in this area, future studies may uncover additional genetic markers that can help refine dental age assessments and improve orthodontic treatment outcomes for Brazilian children and, potentially, populations around the world.

## Conclusion

There was no association between genetic polymorphisms in *ESR1* and *ESR2* and tooth development/dental age in this sample of Brazilian children.
